# Adipocytes express tissue factor and FVII and are procoagulant in a TF/FVIIa-dependent manner

**DOI:** 10.1080/03009734.2019.1645248

**Published:** 2019-08-13

**Authors:** Desirée Edén, Grigorios Panagiotou, Dariush Mokhtari, Jan W. Eriksson, Mikael Åberg, Agneta Siegbahn

**Affiliations:** a Department of Medical Sciences, Clinical Chemistry, Uppsala University, Uppsala, Sweden;; b Department of Medical Sciences, Clinical Diabetes and Metabolism, Uppsala University, Uppsala, Sweden

**Keywords:** Adipocytes, coagulation, FVII, lipolysis, tissue factor

## Abstract

**Background:** Tissue factor (TF) combined with its ligand FVII initiates blood coagulation and intracellular signaling. Obese and type 2 diabetic subjects have increased TF expression in their adipose tissue and an increased risk for thrombotic complications. Here we address the role of TF/FVII on adipocyte functions.

**Materials and methods:** Subcutaneous fat was obtained by means of needle aspiration from healthy volunteers, and adipocytes were isolated after collagenase digestion. 3T3-L1 fibroblasts kept in culture were differentiated into adipocytes by addition of IBMX, dexamethasone, rosiglitazone, and insulin to the media. Proteins and mRNA were analyzed by western blot and RT-PCR. Coagulation activity was determined by a colorimetric FX-assay. Lipolysis was measured as free glycerol using a colorimetric method. Glucose uptake was evaluated by scintillation counting of D-[U-^14^C] glucose.

**Results:** In isolated human primary adipocytes we found expression of TF and FVII. TF expression was confirmed in 3T3-L1 adipocytes, and both cell types were found to be procoagulant in a TF/FVIIa-dependent manner. FXa was generated without FVIIa added to the coagulation assay, and active site-inhibited FVIIa blocked FXa formation, supporting our finding of FVII production by human primary adipocytes. There was no evidence for a role of TF in either lipolysis or glucose uptake in our experimental settings.

**Conclusion:** Human primary adipocytes express active TF and FVII, and the TF/FVIIa complex formed on the adipocyte surface can activate substrate FX. Whether the TF/FVIIa complex conveys signaling pathways leading to biological functions and has any biological activity in adipocytes beyond coagulation remains to be elucidated.

## Introduction

Obesity is an increasing global problem and a major risk factor for development of type 2 diabetes (T2D) (1,2). T2D is associated with a plethora of cardiovascular and thrombotic complications, including atherosclerosis, myocardial infarction, ischemic stroke, and peripheral vascular disease (3–5). Dysregulation of hemostasis is linked to the development of T2D and considered a contributor to its cardiovascular comorbidities. Previous studies report evidence for involvement of both coagulation and fibrinolysis pathways (3,6–8).

Tissue factor (TF), also known as coagulation factor III, is a transmembrane protein and the main initiator of blood coagulation. After vascular injury, TF binds and activates coagulation factor VII (FVII) into FVIIa, and thrombin formation is subsequently initiated. TF is normally expressed on cells outside the vasculature, and activation of the coagulation cascade by aberrant expression of TF promotes thrombotic episodes in patients with a variety of clinical disorders (3,9–12). For instance, obese subjects have increased plasma concentrations of TF and FVIIa and increased expression of TF in their adipose tissue. Moreover, obesity has been linked to increased plasma TF activity (3,13–15). These observations may explain the development of the hypercoagulable state and related complications that are associated with excessive body fat accumulation.

In addition to its role in coagulation, TF/FVIIa also mediates intracellular signaling related to other physiological mechanisms including angiogenesis, migration, tumor growth, apoptosis, and inflammation (16–19). We have previously shown that TF/FVIIa signaling augments cytokine-induced pancreatic beta-cell death, which is proposed to contribute to diabetes (20), while others have coupled TF-mediated signaling to high-fat diet-induced obesity, adipose tissue inflammation, and insulin resistance in mice (12,16,21,22). Obesity is characterized by insulin resistance, and, apart from its role in glucose uptake, insulin also has anti-lipolytic effects (19). Partly due to insulin resistance, obese and T2D individuals exhibit higher lipolysis rates, leading to increased free fatty acids (FFA) concentrations in plasma (23). Moreover, plasma FFA correlates with adipose tissue TF mRNA levels (15), which may indicate a possible role for TF in lipolysis. In the present study, we investigated the impact of TF/FVIIa complex formation on several adipocyte functions in model systems of isolated human primary adipocytes and murine 3T3-L1 adipocytes.

## Materials and methods

### Reagents

Collagenase type II and isoproterenol used in human adipocyte experiments, 3-isobutyl-1-methylxanthine (IBMX), dexamethasone, insulin, and PAR1 (TFFLR) and PAR2 (SLIGRL) agonists used in 3T3-L1 adipocyte experiments were all from Sigma-Aldrich Sweden AB. TNF-alfa, interferon-gamma, and IL-1beta were from R&D Systems, USA. Insulin used in human adipocyte experiments was from Novo Nordisk A/S, Denmark. Human recombinant active site-inhibited FVIIa (FVIIai) and recombinant human and murine FVIIa were kind gifts from L-C Petersen, Novo Nordisk. FX was from Enzyme Research Lab, USA. Rosiglitazone was from Cayman Chemical, USA. TaqMan probes were from Applied Biosystems, USA. The following antibodies were used: GAPDH 14C10, Beta actin 8H10D10, Cleaved caspase-3 D175, pHSL s563, and HSL from Cell Signaling Technology, USA; TF N1C3 from GeneTex USA; TF AF2339 and TF AF3178 from R&D Systems, USA; IRDye 680CW and IRDye 800CW from LI-COR Biosciences, UK.

### Human adipocyte isolation

Isolation of human primary adipocytes from healthy volunteers was essentially performed as previously described (24). Briefly, subcutaneous fat was obtained from the lower abdomen by needle aspiration after dermal local anesthesia with lidocaine (Xylocain, AstraZeneca, Södertälje, Sweden). The study was approved by the Regional Ethics Review Board Uppsala (dnr 2013/183 and 2013/494), and all participants provided written informed consent. Adipocytes were isolated after collagenase type II digestion in Hanks’s medium: medium 199 with Hanks’s salts, L-glutamine, 5.6 mM glucose and HEPES supplement (Life Technologies) supplemented with 4% bovine serum albumin (BSA) and 150 nM adenosine with pH 7.4 at 37 °C in a shaking water bath for 60 min. Following collagenase treatment, adipocytes were filtered through a 250 μM nylon mesh, subsequently held at 37 °C and washed four times in Hanks’s medium before lipolysis and glucose uptake assays were performed.

### 3T3-L1 cell culture, differentiation, and DREAM

3T3-L1 cells (ATCC) were grown to confluence in growth medium: DMEM GlutaMAX with 4.5 g/L glucose and pyruvate supplement (Life Technologies) supplemented with 10% fetal bovine serum (FBS), 100 U/mL penicillin, and 100 µg/mL streptomycin in a humidified chamber at 37 °C and 5% CO_2_. Differentiation was induced by culturing in growth medium supplemented with 2 μM rosiglitazone, 0.5 mM 3-isobutyl-1-methylxanthine (IBMX), 0.25 μM dexamethasone, and 170 nM insulin. After 48 h, medium was changed to growth medium supplemented with 170 nM insulin. Adipocytes were fully differentiated after 8–10 days, and density-based separation followed by re-plating of enriched adipocytes in monolayer (DREAM) was performed, as described by Kajimoto et al. (25).

### MDA-MB-231, HCC1937, and KATOIII cell culture

MDA-MB-231 human breast cancer cells, HCC1937 human breast cancer cells, and KATOIII human gastric carcinoma cells were grown in RPMI medium (Life Technologies) supplemented with 10% FBS, 100 U/mL penicillin, and 100 µg/mL streptomycin in a humidified chamber at 37 °C and 5% CO_2_.

### siRNA transfection in 3T3-L1 adipocytes

DREAMed 3T3-L1 adipocytes were re-plated in 24-well plates in growth medium. Silencer^®^ Select Validated siRNA toward TF and scramble RNA (10 nM Ambion/Life Technologies, USA) were transfected into the adipocytes using Lipofectamine RNAiMAX (Invitrogen AB, Stockholm, Sweden) as described by the manufacturer. Experiments were performed 72 h post siRNA transfection.

### mRNA analysis

Human primary adipocytes were isolated as described and washed three times in phosphate-buffered saline (PBS). Total RNA was extracted using Trizol according to the manufacturer’s protocol (Invitrogen). MDA-MB-231, HCC1937, and KATOIII cells were washed three times in PBS and total RNA isolated using RNeasy Mini Kit according to the manufacturer’s protocol (Qiagen, Hilden, Germany). cDNA was obtained using oligo(dt) (Invitrogen). Reverse-transcriptase PCR (RT-PCR) analyses of TF, FVII, and the housekeeping gene beta-actin were performed on cDNA with Platinum Taq DNA Polymerase as described by the manufacturer (Invitrogen) with 40 amplification cycles. Primers and protocol have previously been published (26). Samples were separated by 1% agarose gel electrophoresis, and bands were visualized by gel red and UV using a Quantity One Flour-S Multi-Imager (Bio-Rad Laboratories, Hercules, CA, USA). Real-time quantitative PCR (qRT-PCR) analyses of PAR2, anti-thrombin III (AT-III), tissue factor pathway inhibitor (TFPI), and the housekeeping gene β2-microglobulin (Assay-on-demand, Applied Biosystems) were performed on cDNA and run on an AbiPrism 7500 (Applied Biosystems).

### Western blot

3T3-L1 and human primary adipocytes were lysed in 2% sodium dodecyl sulphate (SDS) sample buffer supplemented with 5% beta-mercaptoethanol. Proteins were separated on a BOLT mini-gel and transferred to immobilon-FL PVDF membranes (2.5 h at 120 V). The membranes were subsequently blocked 1 h in Odyssey blocking buffer (LI-COR Biosciences) and incubated overnight at 4 °C in Odyssey blocking buffer with primary antibodies diluted 1/1000. After incubation, membranes were washed 3 × 5 min in TBS with 0.01% Tween-20 and incubated 40 min with secondary antibodies (1/10,000 dilution) conjugated to IR-Dyes 680 or 800 and washed as above. Proteins were visualized with the Odyssey Infrared Imaging System (LI-COR Biosciences) and quantified using Odyssey V3.0 software.

### Coagulation activity assay

The coagulation activity assay was performed in isolated human primary adipocytes of a 5% lipocrit in 96-well plates and siRNA transfected 3T3-L1 adipocytes in 24-well plates. Cells were washed three times in HEPES-buffered saline with 0.1% BSA (HBSA). Human primary adipocytes were pre-treated with FVIIai for 5 min or left untreated. Thereafter, cells were incubated with or without 10 nM hFVIIa (murine FVIIa for 3T3-L1 adipocytes), 300 nM FX, and 10 mM CaCl_2_ for 2 h at 37 °C. FXa formation was stopped in HBSA supplemented with 25 mM EDTA and 4 mM chromogenic substrate (S-2765) (ChromogeniX, USA) added for 15 min at 37 °C. Medium from 3T3-L1 cells was moved to 96-well plates for analysis. A standard curve was included in every experiment, ranging from 0.1 pg/mL to 1000 pg/mL of re-lipidated hTF (Siemens, USA), and a trend line was calculated from the standard curve. TF concentration was estimated based on the calculated trend line. Absorbance was read at 405 nm. All experiments were performed in duplicate.

### Lipolysis in human primary adipocyte

The lipolysis assay was essentially performed as described previously (27,28). In brief, isolated adipocytes were in a 2%–3% lipocrit suspension and kept in vials with Hanks’s medium (4% BSA, 150 nM adenosine with pH 7.4) in a gently shaking water bath at 37 °C. Cells were pre-incubated for 30 min with 10 nM human recombinant FVIIa. Lipolysis was evaluated at a basal level and in response to isoproterenol (1 μM for 2 h) with and without insulin (100 μU/mL) pre-incubation for 10 min. Glycerol released to the medium was measured by colorimetric absorbance read at 540 nm with Free Glycerol reagent (Sigma-Aldrich Sweden AB) in a Tecan Magellan plate reader and used as lipolytic index. Duplicate measurements were performed for all conditions.

### Lipolysis in 3T3-L1 adipocytes

Lipolysis assay in 3T3-L1 adipocytes was performed after DREAM in all experiments. Experiments were conducted using the Abcam lipolysis assay kit (ab185433) as described by the manufacturer with adjustments for volumes suitable for 24-well plates. Briefly, cells were washed and kept in buffers supplied in the kit. For the different experiments, 10 nM of mFVIIa or 25 μM of PAR1 or PAR2 agonists was added for 30 min followed by incubation with 5 nM isoproterenol for 3 h. Glycerol released to the medium was measured by colorimetric absorbance read at 570 nm. For lipolysis estimations with long-term cytokine exposure, cells were DREAMed and allowed to settle for 24 h. Thereafter, cells were pre-stimulated with FVIIa (10 nM, 30 min) and subsequently kept for 48 h in growth medium supplemented with a mixture of TNF-alfa (10 ng/mL), IL-1 beta (5 ng/mL), and IFN-gamma (5 ng/mL). Lipolysis assay was thereafter performed as described above.

### Signaling

3T3-L1 adipocytes were DREAMed and allowed to settle overnight. Cells were washed and kept in buffers provided in the lipolysis assay kit. Cells were left untreated or stimulated as follows: 5 nM isoproterenol for 3 h, 1 nM insulin for 10 min followed by 5 nM isoproterenol for 3 h, 10 nM mFVIIa for 3 h, 10 nM mFVIIa for 15 min. After incubation, cells were washed with PBS and western blot performed as described above.

### Glucose uptake in human primary adipocytes

Glucose uptake was essentially performed as previously described (29). Isolated adipocytes, lipocrit 5% were suspended in glucose-free Krebs-Ringer-HEPES (KRH) medium (4% BSA, 150 nM adenosine with pH 7.4) and kept in vials in a gently shaking water bath at 37 °C. Cells were pre-incubated with or without FVIIa (10 nM, 30 min). Cells were left untreated or incubated with insulin (1000 μU/mL, 15 min). D-[U-^14^C] glucose (0.86 μM; GE HealthCare, Buckinghamshire, UK) was added to vials, which were then further incubated for 45 min. Reaction was stopped by transferring adipocytes in suspension to vials on ice, and adipocytes were separated by centrifugation through silicone oil. Glucose uptake was evaluated by triplicate measurements of the adipocyte-associated radioactivity in a beta-counter.

### Statistics

Graphs and statistics were obtained using the GraphPad Prism 7.0a software. Statistical significances were obtained by comparison to corresponding control using Student’s unpaired *t* test (two-tailed). The bars indicate mean + SEM, and *p* ≤ 0.05 was considered statistically significant.

## Results

### TF and FVII are expressed in human adipocytes, and TF can be effectively down-regulated in 3T3-L1 adipocytes

**Figure 1.**
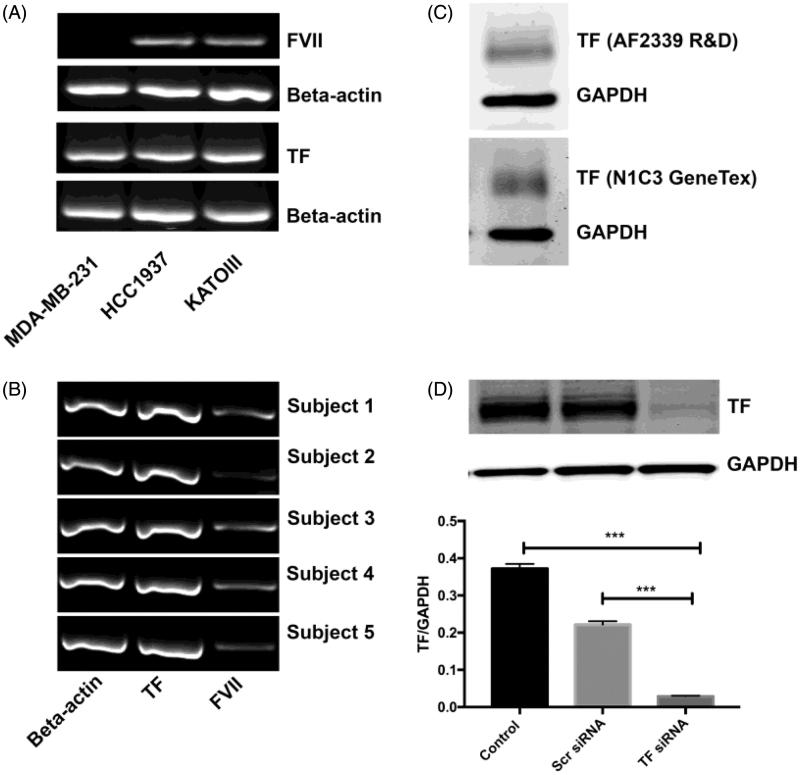
TF and FVII expression in adipocytes. (A) TF and FVII mRNA expression were evaluated with RT-PCR in MDA-MB-231, HCC1937, and KATOIII cells to confirm the method. All cell lines expressed TF. HCC1937 and KATOIII expressed FVII, whereas MDA-MB-231 did not. Beta-actin was included as a positive control. (B) Human primary adipocytes were obtained from healthy volunteers. TF and FVII were expressed on mRNA level, detected with RT-PCR using the same protocol as in (A) (*n* = 5). (C) Human primary adipocytes were obtained from healthy volunteers. TF expression was evaluated on protein level using western blot and two different antibodies towards TF. The figure shows one representative western blot for each antibody; adipocytes in shown experiment were from two different donors. (D) 3T3-L1 adipocytes were DREAMed and cells left untreated or treated with TF siRNA or scrambled siRNA as control for 72 h. Samples were lysed in SDS-sample buffer and protein levels evaluated with western blot (figure shows one representative blot). TF siRNA transfection resulted in a down-regulation of TF with 87% (*p* < 0.01) compared to scr siRNA and 92% (*p* < 0.01) compared to control. *n* = 3. Error bars represent SEM. *** = *p* ≤ 0.001.

First, we wanted to investigate the mRNA expression of TF in isolated human primary adipocytes. It has previously been reported that 3T3-L1 adipocytes and adipose tissue from mice express FVII (30), and therefore we wanted to study the FVII mRNA expression in human adipocytes. To confirm the method, TF and FVII mRNA levels were studied in MDA-MB-231, HCC1937, and KATOIII cells using RT-PCR and previously published primers (26). The cell lines were used as positive controls on the basis of their documented high TF expression and the known ectopic production of FVII by KATOIII cells. MDA-MB-231 cells were used as negative control for FVII expression. All three cell lines expressed TF. HCC1937 and KATOIII cells expressed FVII, whereas MDA-MB-231 lacked FVII expression (Figure 1(A)). Expression of TF and FVII mRNA was then studied in human primary adipocytes from five different subjects using the same protocol. Strong bands were detected for TF, and weaker but distinct bands were detected for FVII in all five subjects (Figure 1(B)). We also performed mRNA analysis for PAR2 and β2-microglobulin, using qRT-PCR, and found that adipocytes express PAR2 (CT mean value: 31.9 ± 0.3 for PAR2 as compared to 19.5 ± 0.2 for β2-microglobulin, *n* = 5; Supplementary Figure 1A, available online). An additional qRT-PCR analysis for AT-III, TFPI, and β2-microglobulin in human primary adipocytes revealed a gene expression of these anti-coagulant factors (CT mean values: AT-III 32.3 ± 0.7, TFPI 26.5 ± 0.3 as compared to β2-microglobulin 20.0 ± 0.3, *n* = 3; Supplementary Figure 1B, available online). TF was studied on the protein level in human primary adipocytes obtained from two donors. Western blot was performed on the lysed adipocytes, and TF expression was evaluated with two different antibodies against TF: TF N1C3 from GeneTex and TF AF2339 from R&D Systems. TF protein was detected with both antibodies (Figure 1(C)). TF was also detected on the protein level in differentiated 3T3-L1 adipocytes using western blot. TF protein levels were effectively down-regulated by siRNA transfection with Lipofectamine on DREAMed 3T3-L1 adipocytes. The TF/GAPDH quota on measured band intensities in TF siRNA-treated cells had a mean of 0.03 compared with scr siRNA-treated cells with a mean of 0.22 and control cells with a mean of 0.37 (*n* = 3). Translated to percentages, TF siRNA treatment down-regulated TF with 87% ± 0.2% as compared with scr siRNA-treated cells (*p* < 0.001) and 92% ± 0.3% compared with untreated control cells (*p* < 0.001) (Figure 1(D)).

### Adipocytes are procoagulant in a TF/FVIIa-dependent manner in both human primary adipocytes and 3T3-L1 adipocytes

**Figure 2.**
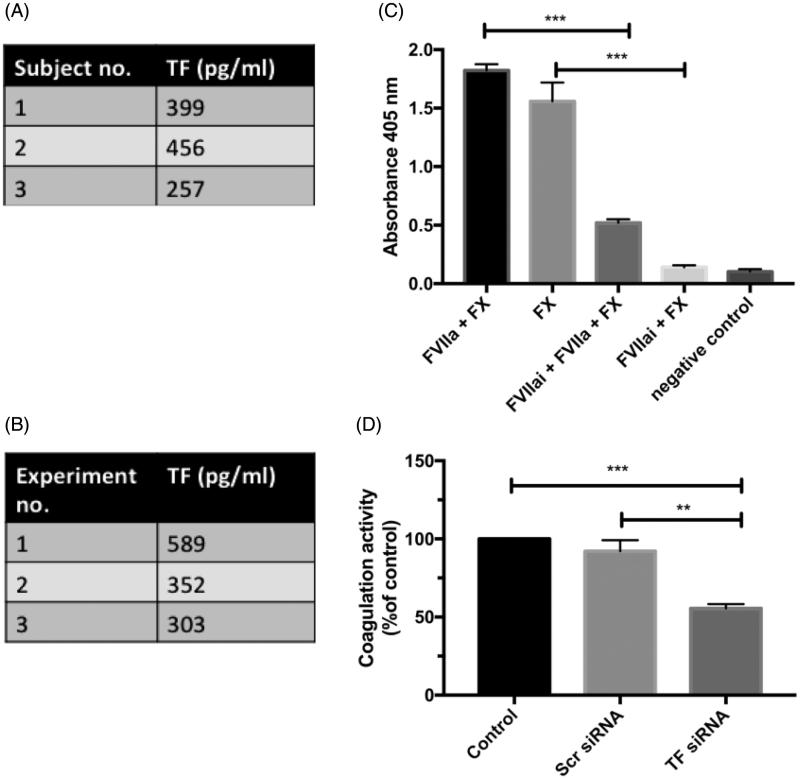
Coagulation activity in adipocytes. (A) Human primary adipocytes were isolated, and coagulation activity was measured as FXa generation after stimulation with 10 nM FVIIa in three distinctive subjects. The table presents active TF in pg/mL based on calculation from standard curve. Three distinctive experiments. (B) Coagulation activity was measured as FXa generation after stimulation of 3T3-L1 cells with 10 nM mFVIIa. The table presents active TF in pg/mL based on calculation from standard curve. Three distinctive experiments. (C) Human primary adipocytes were isolated and pre-stimulated with 100 nM FVIIai for 5 min or left untreated. Incubation was with or without 10 nM FVIIa. Negative control incubated without FVIIa and FX. Graph represents absorbance values. FXa generation was 70% (*p* < 0.001) less in cells treated with FVIIai + FVIIa + FX compared with cells treated with FVIIa + FX and 90% less (*p* < 0.001) in cells treated with FVIIai + FX compared with cells treated with FX. *n* = 3. Error bars represents SEM. (D) TF was down-regulated using siRNA in 3T3-L1 adipocytes, and coagulation activity was evaluated as described. Graph represents absorbance values as percentage of control. FXa generation was 45% (*p* < 0.001) less in cells with down-regulated TF compared with untreated control cells and 40% (*p* < 0.01) less compared with cells treated with scrambled siRNA. *n* = 3. Error bars represent SEM. ** = *p* ≤ 0.01; *** = *p* ≤ 0.001.

Having shown that adipocytes express TF, we continued to study the function of TF by assessing their coagulation activity in a FXa substrate assay. Both human primary adipocytes and DREAMed 3T3-L1 adipocytes were coagulant active in response to FVIIa (Figure 2(A,B), respectively). In human primary adipocytes, there was coagulation activity without addition of FVIIa. Addition of FVIIai together with FVIIa decreased the coagulation activity with 70% in comparison to the group only receiving FVIIa (*p* < 0.01) (Figure 2(C)). TF down-regulation with siRNA transfection using Lipofectamine in 3T3-L1 adipocytes decreased the coagulation activity with 45% compared with untreated adipocytes (*p* < 0.001) and with 40% compared with cells treated with scr siRNA (*p* < 0.01) (Figure 2(D)).

### Lipolysis in human primary adipocytes is not affected by FVIIa stimulation

**Figure 3.**
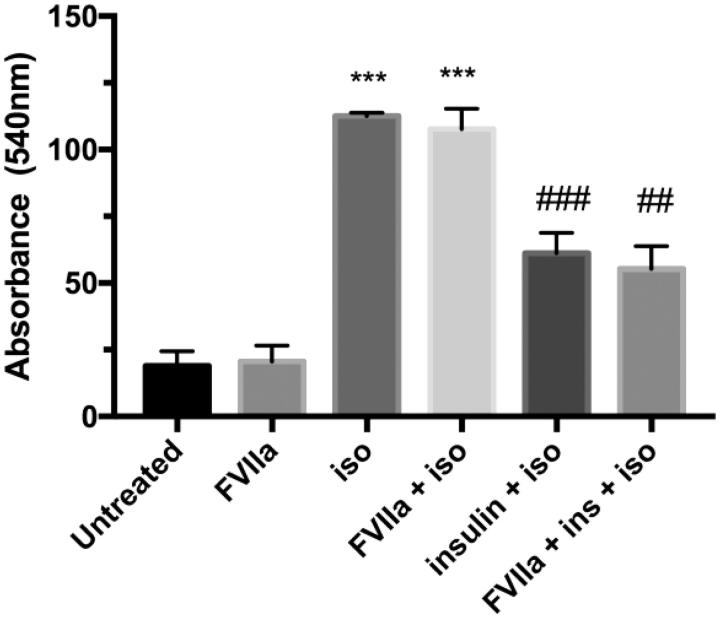
Lipolysis in human primary adipocytes. Adipocytes were isolated from healthy volunteers and kept in a 2%–3% lipocrit suspension in Hanks’s medium in a shaking water bath holding 37 °C. Cells were stimulated with FVIIa (10 nM, 30 min) or left untreated for control. Lipolysis was evaluated on basal level and in response to isoproterenol stimulation (1 uM, 2 h) with and without insulin (100 μU) added 10 min before isoproterenol. Media were collected, and glycerol released to the media was measured by colorimetric absorbance read at 540 nm with Free Glycerol reagent and used as lipolytic index. FVIIa did not alter lipolysis rate in any condition. Isoproterenol increased lipolysis in cells without and with FVIIa compared with untreated cells and FVIIa-treated cells, respectively (*p* < 0.001). Insulin pre-stimulation on isoproterenol-treated samples decreased lipolysis in absence and presence of FVIIa compared with samples only treated with isoproterenol (*p* < 0.001) and samples treated with FVIIa + isoproterenol (*p* < 0.01), respectively. All experiments were performed in duplicate. *n* = 4. Error bars represent SEM. *** = *p* ≤ 0.001 compared to corresponding control group; ## = *p* ≤ 0.01 compared to corresponding isoproterenol-treated group; ### = *p* ≤ 0.001 compared to corresponding isoproterenol-treated group.

Next, we investigated the role of TF in lipolysis in human primary adipocytes. Cells were left untreated or stimulated with 10 nM FVIIa for 30 min. Standard protocols for stimulation and inhibition of lipolysis using isoproterenol and insulin, respectively, were utilized (27,28). Isoproterenol stimulation resulted in increased lipolysis as compared to untreated cells, whereas insulin counteracted the effect of isoproterenol stimulation. FVIIa stimulation did not affect the basal or isoproterenol-stimulated lipolysis, and neither did it alter the anti-lipolytic effect of insulin (Figure 3).

### Lipolysis in 3T3-L1 adipocytes is not affected by FVIIa stimulation, TF down-regulation, and PAR1 or PAR2 agonists

**Figure 4.**
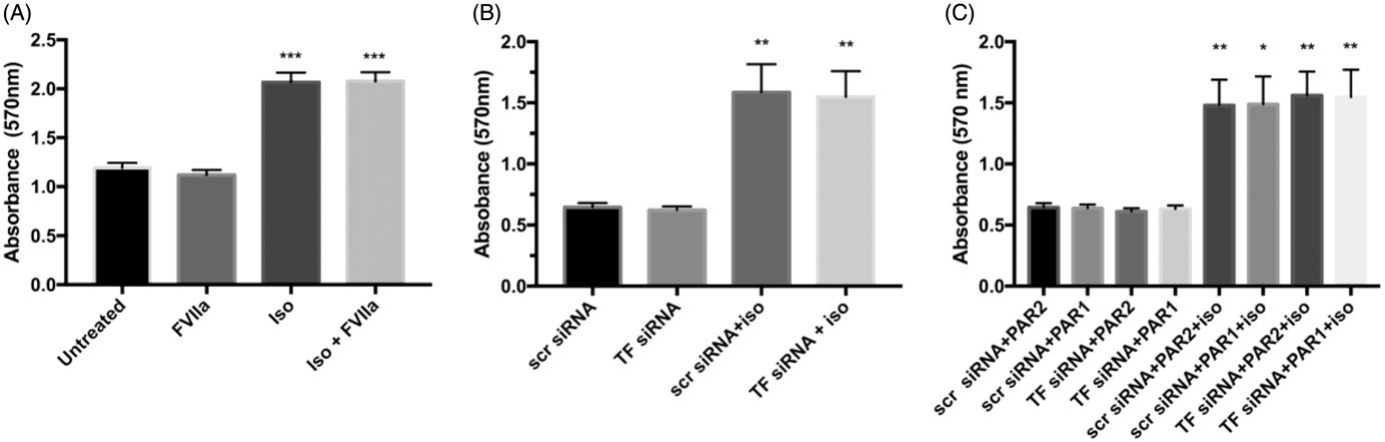
Lipolysis in 3T3-L1 adipocytes. (A) 3T3-L1 adipocytes were DREAMed and incubated for 72 h. After 72 h cells were pre-stimulated with FVIIa (10 nM, 30 min) or left untreated. Lipolysis was stimulated using isoproterenol (5 nM, 3 h), or cells were left untreated. Glycerol released to the media was measured by colorimetric absorbance read at 570 nm with Abcam colorimetric method and assessed as index of lipolysis. FVIIa stimulation does not alter basal or isoproterenol-stimulated lipolysis. Isoproterenol stimulation increases lipolysis in absence and presence of FVIIa compared with untreated and FVIIa-treated cells, respectively (*p* < 0.001), *n* = 4. Error bars represent SEM. (B,C) 3T3-L1 adipocytes were DREAMed and cells treated with TF siRNA or scrambled siRNA as control for 72 h. After 72 h cells were pre-stimulated for 30 min with 25 μM PAR1 or PAR2 agonist or left untreated before lipolysis assay was performed as in A. B and C represent data from the same experimental sets and are directly comparable. (B) TF down-regulation does not alter basal nor isoproterenol-stimulated lipolysis. Isoproterenol stimulation increased lipolysis in cells treated with scr siRNA and TF siRNA compared with respective cells with no isoproterenol stimulation (*p* < 0.01). (C) Neither PAR1 nor PAR2 agonists alter basal nor isoproterenol-stimulated lipolysis (compared with respective cells without PAR agonist stimulation in B), and there is no difference between TF-depleted cells and cells with intact TF. Isoproterenol stimulation increases lipolysis rate in PAR1-treated (*p* < 0.01 for TF siRNA-treated and *p* < 0.05 for scrambled siRNA-treated) and PAR2-treated (*p* < 0.01) cells compared with respective cells with no isoproterenol stimulation. *n* = 4. Error bars represent SEM. * = *p* ≤ 0.05 compared to corresponding control group; ** = *p* ≤ 0.01 compared to corresponding control group; *** = *p* ≤ 0.001 compared to corresponding control group.

To further investigate the possible effects of TF/FVIIa in lipolysis we used 3T3-L1 adipocytes. Isoproterenol stimulation resulted in lipolysis in all experiments similar to the effects seen in the human primary cells. Pre-stimulation with FVIIa (10 nM, 30 min) did not alter the lipolysis rate either at the basal level or in response to isoproterenol (Figure 4(A)).

We further investigated the effect of TF down-regulation using siRNA transfection and also the effect of PAR1 and PAR2 stimulation in lipolysis. Since both the binary complex TF/FVIIa and the trinary complex TF/FVIIa/FXa may trigger signaling through proteolytical activation of PAR1 and PAR2, we stimulated the TF siRNA and scr siRNA-transfected cells with PAR1 and PAR2 agonists or left the cells untreated. There was no difference between TF-depleted cells and cells with intact TF (Figure 4(B,C)). Pre-stimulation with the PAR agonists one by one (25 μM, 30 min) did not alter the lipolysis rate either on the basal level or in response to isoproterenol (Figure 4(C)). Data from Figure 4(B,C) are from the same set of experiments and can be directly compared with one another.

### Long-term cytokine treatment results in lipolysis, but combination with FVIIa stimulation does not alter the lipolysis rate

**Figure 5.**
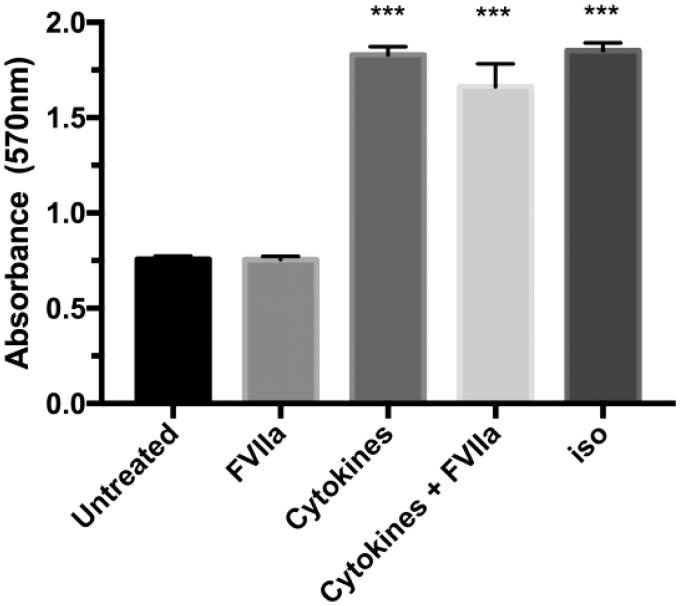
Lipolysis in 3T3-L1 adipocytes after cytokine treatment. Cells were DREAMed and left overnight. On the following day, cells were either left untreated, stimulated with FVIIa (10 nM, 48 h), treated with a cytokine mix (TNF-alfa 10 ng/mL, IL-1-beta 5 ng/mL, and IFN-gamma 5 ng/mL, 48 h), or pre-treated with FVIIa (30 min, 10 nM) before addition of the cytokine mix. Lipolysis assay was performed as described in Methods. FVIIa does not affect glycerol release on basal levels or in combination with cytokines. A 48 h treatment with cytokines (in absence or presence of FVIIa) results in glycerol release from 3T3-L1 adipocytes to the same extent as isoproterenol stimulation (5 nM, 3 h) in contrast to untreated cells (*p* < 0.001). *n* = 4. Error bars represent SEM. *** = *p* ≤ 0.001 compared to corresponding control group.

The adipose tissue of T2D patients and pre-diabetics is likely to be exposed to pro-inflammatory cytokines (6,31). Since cytokines are known to stimulate lipolysis (32), we addressed the question if FVIIa in combination with the pro-inflammatory cytokines TNF-alfa, IFN-gamma, and IL1-beta would exert additional effects on lipolysis. 3T3-L1 adipocytes were DREAMed, allowed to settle for 24 h, and then stimulated with cytokines with or without FVIIa pre-stimulation (10 nM, 30 min) or only treated with FVIIa for 48 h before the lipolysis assay was performed. One group was left untreated and served as a negative control, and one group was stimulated with isoproterenol (5 nM, 3 h) and served as a positive control. Long-term cytokine stimulation of cells led to an increase of glycerol release to the same extent as 3 h of isoproterenol treatment when compared to untreated cells. FVIIa did not alter the lipolysis rate either at the basal level or in response to the cytokine treatment (Figure 5).

### Hormone-sensitive lipase (HSL) is phosphorylated by isoproterenol but unaffected by FVIIa stimulation

**Figure 6.**
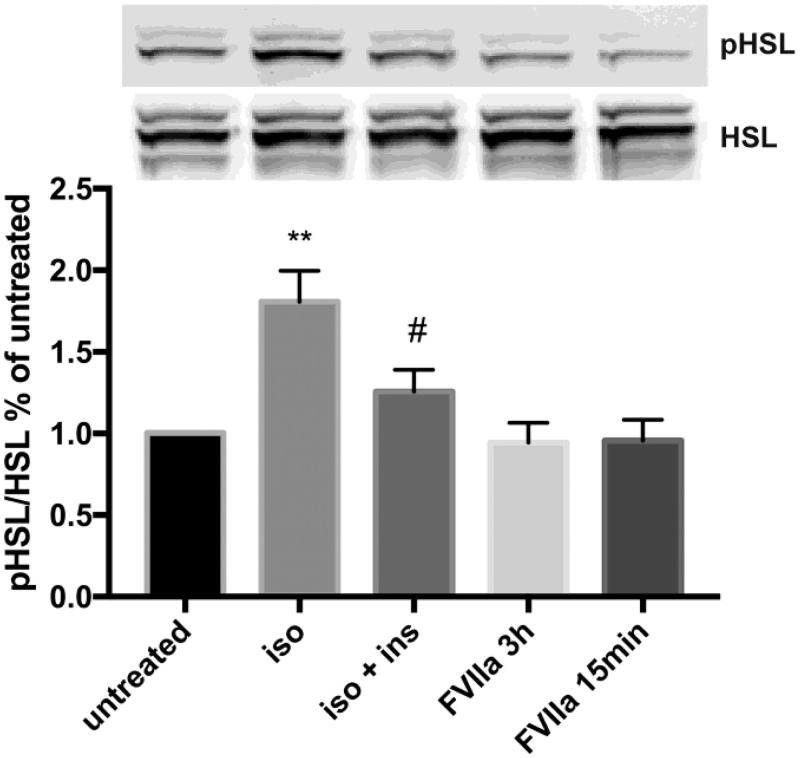
HSL signaling in 3T3-L1 adipocytes in response to isoproterenol and FVIIa. 3T3-L1 adipocytes were DREAMed and left overnight. On the following day, cells were washed and kept in lipolysis assay buffer provided in the Abcam lipolysis kit. Cells were stimulated with 1 nM insulin for 10 min before addition of 5 nM isoproterenol for 3 h, one group only stimulated with isoproterenol and two groups stimulated with 10 nM of mFVIIa for 3 h or for 15 min. One group was left untreated and used as control. Isoproterenol increased phosphorylation of HSL compared to untreated control cells (*p* < 0.01), and insulin counteracts the effect (*p* < 0.05 compared with isoproterenol-treated cells). FVIIa stimulation does not change phosphorylation of HSL compared to untreated control cells in any of the tested settings. *n* = 6. Error bars represent SEM. ** = *p* ≤ 0.01 compared to untreated; # = *p* ≤ 0.05 compared to isoproterenol-treated group.

Lipolysis can be initiated by different ligands and G-protein-coupled receptors, but the signaling pathways eventually converge in the phosphorylation of hormone-sensitive lipase (HSL). Phosphorylated HSL catalyzes the hydrolysis of diacylglycerol to monoacylglycerol that is further hydrolyzed to glycerol and free fatty acids (33). In DREAMed 3T3-L1 adipocytes, isoproterenol increases the phosphorylation of HSL compared with untreated control cells (*p* < 0.01), and insulin counteracts the effect of isoproterenol. There was no difference in HSL phosphorylation in mFVIIa-stimulated cells as compared to that of untreated cells (Figure 6).

### Glucose uptake in human primary adipocytes is not affected by FVIIa stimulation

**Figure 7.**
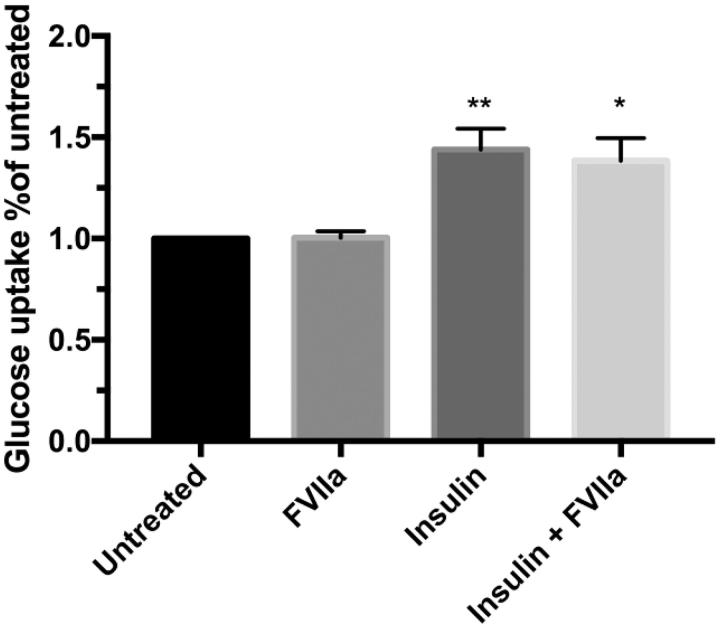
Glucose up-take in human primary adipocytes. Adipocytes were isolated from healthy volunteers and kept in a 10% lipocrit suspension in KRH media (4% BSA, 150 nM adenosine with pH 7.4). Cells were stimulated with FVIIa (10 nM, 30 min) or left untreated for control. Glucose uptake was evaluated on basal level and in response to insulin (1000 µU/mL, 15 min). D-[U-^14^C] glucose was added and incubated 45 min. Reaction was stopped on ice and adipocytes separated by centrifugation through silicone oil. Glucose uptake was evaluated by measuring the adipocyte-associated radioactivity in a beta-counter. Graph represents counts per minute as percentage of untreated. FVIIa did not alter glucose uptake on basal level or in response to insulin. Insulin increased glucose uptake in absence (*p* < 0.01) and presence of FVIIa (*p* < 0.05) compared with untreated and FVIIa-treated cells, respectively. *n* = 4. Error bars represent SEM. All experiments performed in duplicate. ** = *p* ≤ 0.01 compared to untreated; * = *p* ≤ 0.05 compared to FVIIa-stimulated group.

Lastly, we investigated the effect of TF/FVIIa on glucose uptake in human primary adipocytes. Isolated cells were left untreated or pre-treated with FVIIa and incubated with or without insulin (1000 µU/mL). Insulin stimulation increased glucose uptake (*p* < 0.01), but pre-treatment with FVIIa did not alter basal or insulin-stimulated glucose uptake (Figure 7).

## Discussion

In the present study we showed that both isolated human primary adipocytes and 3T3-L1 adipocytes express TF and are procoagulant. We also showed that isolated human primary adipocytes express FVII. However, TF/FVIIa signaling pathways are not involved in the lipolysis or glucose uptake of these cells.

Previous studies have demonstrated that human adipose tissue and murine adipocytes express TF (15,22,34), but to the best of our knowledge this is the first study providing evidence that human primary adipocytes themselves have high expression of TF. The most well-known function of TF/FVIIa is the initiation of coagulation (35). Both human primary adipocytes from the subcutaneous adipose depots and 3T3-L1 adipocytes clearly proved able to form FXa in response to FVIIa stimulation. It has been suggested that the anatomical location of adipose tissue predisposes to different types of thrombotic event after injury (36). Subcutaneous adipose tissue (SAT) is associated with venous thrombosis and shows stronger relationship with functional measurements of hypercoagulability after injury as compared to visceral adipose tissue (VAT) (36). Many studies have pointed to the association between adipose tissue and hypercoagulability (37, and references therein), and the present study furthers this association by providing evidence for human as well as murine adipocytes to initiate coagulation activity. In a study comparing proteomics in VAT from pre-obese non-diabetics with pre-obese T2D patients it was found that AT-III was increased in T2D subjects (38). In our study we used primary adipocytes from the subcutaneous depots of healthy volunteers and found mRNA expression of both AT-III and TFPI in these adipocytes. Even though SAT and VAT have distinct characteristics it cannot be ruled out that T2D would increase the AT-III expression in SAT as well. Most likely, the presence of AT-III and TFPI in SAT suppresses the coagulation when initiated, and this may explain why there are not even more clinical events in the form of thrombosis.

When we performed the coagulation assay in human primary adipocytes without adding FVIIa we still recorded FXa formation at almost the same rate as when FVIIa was added. Addition of active site-inhibited FVIIa (FVIIai) to isolated human primary adipocytes significantly reduced the generation of FXa. FVIIai is a derivative of FVIIa where the catalytic site is rendered inert (39). FVIIai retains its ability to bind TF, and a conformational change in the protein gives it an increased affinity and prolonged dissociation half-life as compared to FVIIa. In contrast to TF/FVIIa, the TF/FVIIai complex does not induce coagulation activity. We propose that the dampening effects by FVIIai on the procoagulant activity, induced in the absence of recombinant FVIIa, is explained by our finding that human primary adipocytes have their own expression of FVII. The predominant source of FVII under normal physiologic conditions is hepatocytes, but they are not the only known FVII-producing cells. During inflammation and atherosclerosis it has been found that monocytes/macrophages express FVII (40–42). Ectopic expression of FVII has also been found to be frequent in various cancers, where the signaling promotes cell migration and invasion activities (26). It has previously been reported that 3T3-L1 adipocytes and murine adipose tissue express FVII (30), but we now provide evidence for FVII expression in isolated human primary adipocytes as well.

The coagulation process *in vivo* is readily initiated at picomolar levels of FVIIa, and only few TF molecules are needed on the cell surface for this step (43,44). About 90% TF protein down-regulation efficiency in the 3T3-L1 cells resulted in reduction of the coagulation activity by 45%. The discrepancy between TF down-regulation and the remaining TF coagulation activity is best explained by the described potency of the coagulation system.

Lipolysis is typically initiated by activation of beta-adrenergic receptors on the surface of the adipocyte. This indirectly leads to increased production of cyclic adenosine monophosphate (cAMP) and protein kinase A (PKA) and, further downstream, to activation of lipases (33). One of these lipases is hormone-sensitive lipase (HSL) that needs to be phosphorylated for lipolysis to occur. Insulin binding to the insulin receptor results in downstream activation of PI3K/AKT that inhibits PKA activity, resulting in decreased lipolysis (33). TF/FVIIa signaling is able to activate both PI3K/AKT signaling, and activation of PAR2 can cause PKA activation (45,46). Using quantitative real time PCR (qRT-PCR), we found human primary adipocytes to express PAR2 (Supplementary Figure 1A, available online). We therefore speculated that TF/FVIIa signaling could affect either the pro- or the anti-lipolytic pathway. However, we failed to see any effect of TF/FVIIa signaling on lipolysis in any of the experiments performed. The endogenous FVII produced by the human primary adipocytes was enough to stimulate coagulation activity to almost the same level as when the cells were treated with 10 nM FVIIa. However, coagulation is initiated at picomolar concentrations of FVIIa, whereas TF/FVIIa signaling has been shown to be dose-dependent up to 50 nM concentration of FVIIa (43,44,47). It is therefore unlikely that the FVII produced by the adipocytes would be enough to stimulate signaling to such an extent that it would mask any effects of the FVIIa stimulation in our lipolysis experiments. If the lack of effect on lipolysis by FVIIa stimulation did depend on a saturation of FVIIa, there should have been an effect on lipolysis in the experiments with TF knock-down.

Addition of the cytokine mix for 48 h increased the glycerol release to the same extent as 3 h of isoproterenol stimulation, but FVIIa had no additional effect. No increase in HSL phosphorylation due to the cytokine treatment was recorded by western blot analyses performed on lysates from these experiments, but we did notice an increase in cleaved caspase 3 in the cytokine-treated group (Supplementary Figure 2, available online). The western blot data suggest that the increased glycerol release observed in the cytokine-treated groups might be caused by apoptosis rather than lipolysis. However, it has previously been reported that IL-1beta and TNF-alfa can induce lipolysis and that TNF-alfa in adipose tissue contributes to insulin resistance, which indirectly further increases lipolysis (reviewed in [32]). In perspective of these results, it is possible that the long-time stimulation in our experiments leads to a miss of the phosphorylation of HSL or that lipolysis in this setting occurs via some other signaling pathway. Regardless of the reason for the increased glycerol release in the cytokine-stimulated groups, FVIIa addition had no effect.

We have previously shown that TF/FVIIa signaling affects glucose-stimulated insulin secretion in human pancreatic islets, and thus there is a connection between TF/FVIIa and glucose signaling (20). In the present study, we investigated whether FVIIa stimulation would affect the uptake of glucose in human primary adipocytes. However, TF/FVIIa had no effect on glucose uptake in the adipocytes. Together with the lipolysis results, this does not support a direct interaction between coagulation activity and insulin action in adipose cells.

In conclusion, we have demonstrated that human primary adipocytes and 3T3-L1 adipocytes express active TF, human primary adipocytes express FVII, and, moreover, we found that the TF/FVIIa complex formed on the adipocyte surface can activate the macromolecular substrate FX. In comparison, TF/FVIIai formed on human adipocytes and depletion of TF in 3T3-L1 adipocytes significantly decreases FXa formation.

Quite in contrast, TF/FVIIa did not exert biologically relevant effects on lipolysis or glucose uptake, nor were there any effects of FVIIa when added in combination with cytokines. Whether the TF/FVIIa complex affects signaling pathways in adipocytes leading to biological activity beyond initiation of coagulation remains to be further investigated.
